# Effectiveness of a multicomponent intervention to reduce social isolation and loneliness in community‐dwelling elders: A randomized clinical trial

**DOI:** 10.1002/nop2.1277

**Published:** 2022-06-24

**Authors:** José Hernández‐Ascanio, Luis Ángel Perula‐de Torres, Manuel Rich‐Ruiz, Josefa González‐Santos, Juan Mielgo‐Ayuso, Jerónimo González‐Bernal, ASyS Study Collaborative Group, Ana María Roldán‐Villalobos, Carlos Pérula ‐ de Torres, Rodrigo Fernández‐Márquez, Caridad Dios‐Guerra, Manuela Urbano‐Priego, Silvia Luna‐Morales, Rafaela Muñoz‐Gómez, Miguel Á. Gómez‐Torres, Ángeles Pastor‐López, Mª José Ibáñez‐Fernández, Mª Dolores Aguilera‐López, Antonio González‐Delgado, Diego Garrido‐Gálvez, Trinidad Romero‐Sánchez, Inmaculada Guzmán‐Castilla, Mª Dolores Maestre‐Serrano, Mari Paz Gutiérrez‐Martín, Eva Sánchez‐Cañete, Yolanda Sánchez‐Palomo, Manuela Rodríguez‐Priego, Ana Mª Pérez‐Trujillo, Santiago Cruz‐Velarde, Sonia Calero‐Sánchez, Rocío Hidalgo‐Navarro, Fco. Javier Ruiz‐Moruno, Mª Reyes Martínez‐Guillén, Antonia Domínguez‐Ramírez, Mª Ángeles Ortega‐Osuna, Paulina Menéndez‐Sagrado, Montserrat Jabalera‐Ramírez, Fernanda Casado‐Salinas, Carmen Mª Mirás‐García, Pilar Conde‐Moya

**Affiliations:** ^1^ Maimonides Institute for Biomedical Research (IMIBIC) University of Cordoba (UCO) Reina Sofía University Hospital (HURS) Córdoba Spain; ^2^ Family and Community Medicine Unit of the Cordoba‐Guadalquivir Health District Córdoba Spain; ^3^ CIBER on Frailty and Healthy Ageing (CIBERFES) Health Institute Carlos III Madrid Spain; ^4^ Universidad de Burgos Burgos Spain

**Keywords:** community‐dwelling, elders, loneliness, nurses, nursing, quality of life, social isolation

## Abstract

**Aims:**

To assess the effect of a multicomponent intervention on reducing social isolation and loneliness and improving the quality of life in community‐dwelling older adults.

**Design:**

A cluster‐randomized controlled clinical trial.

**Methods:**

A total of 56 older adults participated in the control group and 63 older adults in the experimental group. The intervention consisted of 6 home‐based face‐to‐face sessions, intercalated with 5 telephone calls and was conducted by nursing students and volunteer staff with experience in the subject. The study was conducted between April 2018 and December 2019. In terms of statistical analysis, several procedures were carried out: a statistical analysis per protocol and intention to treat, considering isolation, loneliness and quality of life as endpoints; a comparison of paired means, to analyse the behaviour of the outcome variables at baseline and the end of the study; and finally, a binary logistic regression analysis, considering social support as a dependent variable.

**Results:**

The study results do not show the effectiveness of the modified CARELINK programme, analysed as a whole, on the decrease in social isolation or loneliness or the improvement in HRQL. However, a detailed analysis of the behaviour of some of the variables during the study indicates some results that deserve to be commented on. Comparing the mean confidential support scores between the experimental and control group at the initial and final stages shows significant differences in the analysis by protocol, and close to statistical significance in the analysis by intention to treat. Comparing the paired means obtained in the experimental group, an improvement in emotional loneliness scores was found. Finally, the variables associated with the social support of the subjects at the end of the follow‐up period were as follows: having people who help them and mobility.

**Conclusion:**

Although the results obtained do not allow us to affirm that the intervention programme is effective, these same results point to improved confidential support and emotional loneliness in older adults participating in the intervention. Having people to help them and a greater degree of mobility are factors favouring the decrease in social isolation.

**Impact:**

This study suggested that modified CARELINK, a multicomponent intervention performed by trained volunteers, could improve confidential support in community‐dwelling older adults. It also reports the importance of considering the level of mobility and support networks as determinants of the improvement caused by the intervention.

## 1 INTRODUCTION

Social isolation and unwanted loneliness are complex and interrelated phenomena (Doblas & Díaz Conde, 2018) that have raised an increasing concern in multiple areas ranging from health care to politics to social and economic areas (Freedman & Nicolle, 2020; Leigh‐Hunt et al., 2017).

The reasons for this interest are clear. On the one hand, many studies have highlighted the adverse effects that social isolation and unwanted loneliness have on the health and quality of life of subjects (Christiansen et al., 2016; Holt‐Lunstad et al., 2015; Leigh‐Hunt et al., 2017), producing a clear deterioration of both.

On the other hand, important economic effects have been described, such as increased use of healthcare services (Hand et al., 2014).

In addition, it is necessary to bear in mind that these are frequent phenomena in vulnerable populations: Older people, living alone, with few support networks, low education levels and bad financial conditions (Bosma et al., 2015; Doblas & Díaz Conde, 2018; Hernán Montalbán & Rodríguez Moreno, 2017; Lasgaard et al., 2016; Nicolaisen & Thorsen, 2014; Pikhartova et al., 2016).

If we bring together the growing dimensions of this type of population (older, living alone and bad financial condition), these phenomena have become a health problem and a global social and public health problem in Western countries (Cacioppo & Cacioppo, 2018).

In this context, it is not surprising that there is a significant increase in very heterogeneous intervention proposals whose objectives aim to prevent these situations and reduce the negative impacts (Masi et al., 2011; (O'Rourke et al., 2018).

### 1.1 Background

Concerning the contents of the interventions, we find a large diversity of activities such as creative projects of art, painting and poetry (Swindells et al., 2013); programmes of face‐to‐face and telephone accompaniment (Kime et al., 2012); programmes with technological components (Hagan et al., 2014); exercise‐based programmes (Tse et al., 2014); programmes offering gardening/horticulture workshops (Brown et al., 2004; Chen & Ji, 2015) or animal‐assisted therapy‐based programmes (Vrbanac et al., 2013; Banks & Banks, 2002; Greaves & Farbus, 2006).

Despite this, little is known about the scope of the interventions available, their effectiveness and the factors contributing to their success (Cattan et al., 2005; Oliver et al., 2006; Valtorta & Hanratty, 2012). In recent years, a series of systematic reviews have been performed, and their primary objective has been to evaluate the effectiveness of different interventions in the field of social isolation and loneliness in older adults (Cattan & White 1998; Cattan et al., 2005; Cohen‐Mansfield & Parpura‐Gill, 2007; Dickens, Richards, Greaves et al., 2011; Dickens, Richards, Hawton, et al., 2011b; Findlay 2003; Frank et al., 2016; Hagan et al., 2014; O´Brien et al., 2014). The analysis of the studies included in these reviews suggests that the more effective interventions are those: (1) that are developed within the framework of a theoretical basis (vs. those interventions that do not have a clear theoretical basis); (2) that are group interventions (vs. individual format) offering social activities and support (vs. visits or interventions via the internet) and (3) in which the older adults are active participants (vs. non‐participatory activities).

The Carelink intervention strategy is characterized by being a model of intervention based on theoretical and epistemological principles of the nurse discipline, of individual nature, in which a combination therapy of reminiscence, discussion of news, speaking during exercise, coaching and modelling are applied, in which the elders are active participants (Nicholson & Shellman, 2013). In addition, it should be highlighted that one of the key aspects of this intervention is that it is based on the specific needs of each individual, and uses the resources were existing in the community (Saito et al., 2012).

However, the studies included in the systematic reviews were heterogeneous and with a medium‐high risk of biases (Dickens, Richards, Greaves et al., 2011; Dickens, Richards, Hawton, et al., 2011b; Frank et al., 2016; Gardiner et al., 2018); and they often show inconsistent results, preventing conclusive evidence.

Therefore, this study aims to answer the question: What is the effectiveness of an adapted model of CARELINK intervention (Nicholson & Shellman, 2013) to reduce the conditions of social isolation and loneliness in community‐dwelling older adults? To answer this question, we propose the implementation of the present clinical trial.

## 2 THE STUDY

### 2.1 Aims

(1) To assess the effect of a multicomponent intervention on reducing social isolation and loneliness and improving health‐related quality of life (HRQOL), and (2) to identify the associated factors in the improvement of social isolation and loneliness in non‐institutionalized older adults.

### 2.2 Design

A cluster‐randomized, two parallel groups, multicenter, controlled clinical trial was performed. The study was conducted between April 2018 and December 2019. The study protocol was published in January 2020 (Hernández‐Ascanio et al., 2020) and was recorded on ClinicalTrials.org (NCT03345862).

### 2.3 Participants

The clinical study was performed in the network of Primary Care Centers of the Health District of Córdoba and Guadalquivir, of the Andalusian Health Service (Spain).

The inclusion criteria for the participants were as follows: age 65 years or older, community‐dwelling (not institutionalized), with social isolation (score less than 32 on the Duke‐University of North Carolina (UNC) Functional Social Support Questionnaire (DUFSS)) (Bellón et al., 1996).

The exclusion criteria for participating in the study consisted of presenting at least one of the following indicators: (1) severe cognitive impairment (8–10 errors in Pfeiffer's Short Portable Mental Status Questionnaire) (Martínez de la Iglesia et al., 2001); (2) clinical diagnosis of dementia; (3) difficulty responding to the measurement scales by language barriers, (4) physical, mental or legal disability; or (5) not providing consent for participation in the study.

A sample size of 57 subjects per group was set to detect a 3‐point increase in the DUFSS or a 0.9‐point decrease in the Loneliness Jong‐Gierveld scale after the intervention. Power was 80%, the confidence level was 95%, the sample size ratio between the control group and the experimental group was 1:1, and the estimated percentage of losses or withdrawals in the follow‐up was 20%.

The subjects participating in the study were recruited by consecutive sampling, through 32 healthcare professionals (3 general practitioner residents, 9 general practitioners and 20 nurses), from 13 healthcare centres. A random allocation was performed with a ratio of 1:1 depending on the healthcare center where the recruitment was conducted (9 centres for the experimental group with 19 researchers and 57 patients: 3 patients/researcher; and 8 centres for the control group with 14 researchers and 57 patients: 4 patients/researcher). This random assignment was performed centrally using the EPIDAT program, version 3.1.

### 2.4 Intervention

A multicomponent systematized intervention was conducted in the experimental group, while only a follow‐up of the situation was conducted in the control group.

The overall aim of the intervention conducted on the experimental group is to stimulate social integration and to move the older adults towards the goal of renewed socialization. The contents of the intervention and the component that is pursued with each one are summarized in Table 1.

**TABLE 1:** Components and actions of the adaptation of the CARELINK programme

Component	Actions
Communication and social contact	Action 1: Conversation while performing daily activities Action 2: Discussion about the news
Feelings of competence and self‐control	Action 3: Evocation and reminiscence of motivating activities or desirable participation contexts. Action 4: Identification of causes, factors related to the loneliness and isolation situation. Action 5: Planning of activities that the subject is able to perform. Action 6: Training in control and coping skills. Action 7: Positive reinforcement of achievements and motivation to achieve the following accomplishments.
Participation in social activities	Action 8: Information on social community resources favouring participation and connection with other people

In the conversation, while performing daily activities, the intervention agent and the older adult performed objective‐oriented exercises while discussing the social aspects of health. The idea was to perform a common activity to focus attention on the activity and, in this way, the social discussion would flow naturally.

The discussion about the news aimed to motivate the interest of elders in the environment and “outside life,” with different settings (from the closest to the most general environment). In this way, the intervention agent and the older adult spoke of news from both the elder's close environment and news that appeared in the media (newspaper, television, etc.).

In reminiscence therapy, older adults discussed positive aspects of their lives when they believed they were more socially integrated. The aim of this technique was to allow the person to remember the value and relative ease of social engagement and, in this way, to get older adults to participate socially as they did in the past.

In this sense, coaching was used to help older adults achieve social integration. As older adults tried to achieve or even achieved their social goals, they were encouraged to continue and they received positive feedback. Modelling focussed on sharing personal experiences related to appropriate social behaviour, with the aim of encouraging older adults to emulate such social experiences.

For identification of causes and planning of activities aimed at social engagement, the intervention agent and the older adult sat face to face and discussed social isolation. These talks addressed the causes of the elder's isolation but also sought to find new solutions. In addition, information on social community resources was provided to the older adults to favour their participation and networking with other people.

The ability to alter the care plan for each visit made the older adults feel that they were leading the visit, which supported their empowerment.

This intervention was an adaptation of the CARELINK intervention programme (Hernández‐Ascanio et al., 2020) through a clinical sociology approach. The modification performed resulted from an initial study aiming to assess the feasibility of the intervention to be tested in the clinical practice of our primary care services.

The modification consisted of an adaptation of the number, duration and frequency of sessions, but no changes in the content of sessions were performed. The modified intervention comprises 6 home sessions of at least 30 min and 5 telephone calls of at least 20 min. Face‐to‐face and telephone sessions were interspersed over 4 months (16 weeks), depending on the individual characteristics of each person. The first contact, oriented on performing an initial assessment, defining objectives, and creating a trust relationship, was 1 hr long and was performed face‐to‐face.

Furthermore, difficulties in a first pilot study conditioned that this study was finally conducted by volunteer staff, composed of students of the degree of nursing from the School of Medicine and Nursing of the University of Córdoba and volunteers of non‐governmental organizations related to the subject.

A total of 13 nursing students (2 of them dropped out) and 17 volunteers from social organizations (8 of them dropped out) participated in the project as intervention agents.

All intervention agents had previous (non‐professional) experience in the health and social field, but not specifically in intervention on social isolation. Therefore, a training activity was designed in which all intervention agents participated. This activity consisted of 4 3‐hr sessions, in which they acquired the necessary skills for the implementation of the programme. This training combined content presentation and role‐play. Moreover, the intervention agents received advice and supervision from two members of the research team who supported the implementation of the intervention throughout the whole process.

### 2.5 Data collection

The follow‐up time for each patient was 6 months. The people in the intervention group were evaluated at three different time points: at the baseline, before the onset of the intervention (T1), a second‐time point at the end of the intervention (4 months after the onset of the intervention) (T2), and a third final time point 2 months after the end of the intervention (T3). In the control group, only two measurements were performed, at baseline (T1) and 6 months after this measurement (T3). Both the experimental and control groups recorded losses (relocation, death, etc.) and withdrawals (refusal to continue, …).

The baseline variables of the study were as follows: demographic characteristics (age, sex, marital status, living alone, level of education, last occupation held, financial condition, and financial help), health status (heart rate, blood pressure, weight, height, Body Mass Index [BMI], mobility level, functionality, and chronic morbidity), attending healthcare centres in the last 3 months (number of consultations, type of consultation and type of professional who attended them), and factors related to social isolation and loneliness (support networks, unmet expectations in their relationship with family and friends, depression and coping skills). In addition, social isolation, loneliness and health‐related quality of life (HRQOL) (end‐point or outcomes) were measured at all time points (including at baseline).

The primary outcome was social isolation. The DUFSS was used to measure this social isolation. This questionnaire considers the opinion on the availability of other people to provide assistance in difficulties, skills in social relationships, and empathic and emotional communication. The scale has two dimensions: confidential and affective. It consists of 11 items that are answered using a scale of 1 to 5 points (limits: 11–55), where high scores represent larger social support than the lower scores. A score of 32 or above indicates normal support, whereas less than 32 indicates low perceived social support (Bellón et al., 1996; Ayala et al., 2012; de la Revilla et al., 1991).

In addition to social isolation, loneliness and quality of life were measured as constructs potentially influenced by the intervention. The Jong‐Gierveld Loneliness scale was used to measure loneliness; this scale values the subjective individual perception of social participation or isolation in the elderly population. Two components are distinguished: emotional loneliness and social loneliness. It consists of six items in its abbreviated version, scored on a scale from 0 to 2 but subsequently recoded as dichotomous (0 or 1). The overall scale indicates a larger sense of loneliness with high scores (limits: 0 to 6). It was validated for Spain in a population aged 60 years and older by Ayala et al. (2012). EuroQol‐5D (EQ‐5D) was used to assess HRQOL. This scale includes 5 dimensions. Each response is coded as 1, 2 or 3. This data establishe the health status of the individual by a number of 5 digits (one for each dimension studied). With this system, 243 different theoretical health states can be codified. The second part of the EQ‐5D is a Visual Analogue Scale (VAS) ranging from 0 (worst imaginable health status) to 100 (best imaginable health status). In the VAS, the subject should mark on a vertical line the point that best defines their global health status today. The use of the VAS provides a complementary score to the descriptive system of self‐assessment of the health status mentioned above. Its psychometric properties have been validated in both the general population and groups with diseases, and an index of preference values for the health status, obtained in a Spanish population (Badia et al., 1999), is available.

### 2.6 Data analysis

A statistical analysis per protocol (in which the statistical analysis included those subjects who completed the study) and intention to treat was conducted; in the latter, the last observation data recorded were due to the withdrawal. A comparison of paired means was carried out to analyse the behaviour of the variables of outcome (social isolation feeling of loneliness) between the baseline and the end of the study. The Chi‐square test or Fisher's exact test, when applicable, and Student's t‐test or Wilcoxon test (*p* < 0.05, bilateral contrasts) were used. ANOVA test was used for repeated measurements to test the differences in the endpoints of the study between the experimental group and the comparison control group, adjusting for baseline values. The normality was assessed with the Kolmogorov–Smirnov test.

A binary logistic regression analysis was performed considering social support as a dependent variable (low social support: Duke‐UNC score ≤ 32, vs. normal social support, score above 32) to check the effect of the intervention on social isolation, to identify the associated or determining factors and to control possible confounding factors. Ordinal variables were treated as dummy variables. The goodness of fit of the logistic regression model was evaluated using the Hosmer–Lemeshow test. The modelling strategy consisted of starting with a maximum model with all the independent variables presumably predictive or confounding and removing, step by step, those variables with a *p* ≤ 0.05, until the most parsimonious model was achieved. The variables introduced in the maximum model were as follows: group, age, sex, marital status, living with others (living alone or accompanied), education level, financial condition, financial help received, social support network (has people who help them), medical demand in primary care, demand for a nurse in primary care, domiciliary health care, nursing outcomes classification (NOC) mobility, NOC depression, NOC coping, baseline social support, feeling of loneliness, state of health (Euroqol‐5D), and number of chronic diseases. The analysis was performed using the SPSS v.22 statistical package.

### 2.7 Validity and reliability/rigour

Prior to the intervention, a guide for the overall process for a visit was distributed to participants, and a 4‐hr educational and training plan was conducted among the intervention agents of the experimental group. In addition, on‐demand consulting sessions (from these agents) were provided with professionals of reference. The monitoring, mechanization and processing of the data were performed by a single person in charge of monitoring the study.

## 3 RESULTS/FINDINGS

**FIGURE 1:**
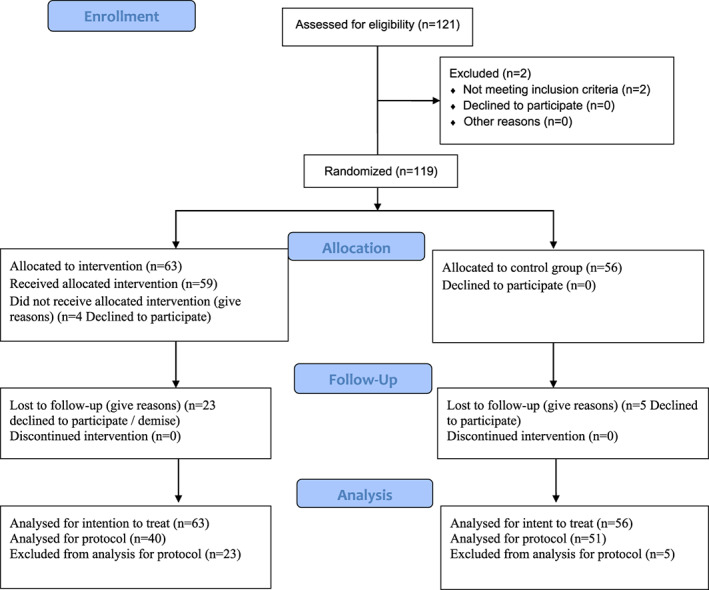
Flowchart of the participants in the trial

Although it was initially intended that 114 patients would be recruited for the study, it was possible to recruit 121 subjects, 92 of them completed the study. A total of 4 subjects from the control group dropped out the study, while 23 subjects from the experimental group dropped out or died (Figure 1). Some of the difficulties identified in this regard are addressed in the qualitative study complementary to the present article.

### 3.1 Characterization of the sample at baseline

First, it should be noted that no statistically significant differences have been found in the analysis of baseline conditions between the experimental and control groups (Tables 2 and 3). However, differences have been found in two variables: (a) the availability of formal or informal support networks, because the experimental group reported having more support in telecare (20.6%) than the control group (5.4%) or unpaid support service and others (4.8% in the experimental group and 0% in the control group; *p* < 0.001), and (b) the expectations coverage regarding family and friends because the experimental group has higher levels of dissatisfaction than the control group (67.8% vs. 46.2%, respectively; *p* = 0.021).

**TABLE 2:** Comparison between control and intervention groups at baseline (depending on socio‐demographic characteristics)

Variables	Group				Total (*N* = 119), Mean ± SD (range)	
	Control (*N* = 56), Mean ± SD		Experimental (*N* = 63), Mean ± SD			
	*N*	%	*N*	%	*N*	%
Age (years)	82.91 ± 6.86		80.79 ± 5.38		81.79 ± 6.27 (66–94)	
Sex						
Male	13	23.2	15	23.8	28	23.5
Female	43	76.8	48	76.2	91	76.5
Marital status						
Married	6	10.7	14	22.2	20	16.8
Separated	4	7.1	5	7.9	9	7.6
Single	6	10.7	4	6.3	10	8.4
Widowed	40	71.4	40	63.5	80	67.2
Living with others at home						
Spouse or partner	6	10.7	11	17.5	17	14.3
Without a partner, but forming the nuclear family with a child or other family member	4	7.1	5	7.9	9	7.6
Living alone	46	82.1	46	73.0	92	77.3
Without a partner, but lives with other non‐relatives	0	0.0	1	1.6	1	0.8
Education level						
No studies	24	42.9	23	36.5	47	39.5
Incomplete primary school	17	30.4	24	38.1	41	34.5
Primary school graduate	10	17.9	16	25.4	26	21.8
Secondary school	5	8.9	0	0.0	5	4.2
Last occupation held						
External	35	62.5	45	71.4	80	67.2
Internal	20	35.7	18	28.6	38	31.9
Unknown	1	1.8	0	0.0	1	0.8
Financial condition (difficulties)						
Rarely or almost never	32	57.1	34	54.0	66	55.5
From time to time	12	21.4	14	22.2	26	21.8
Often	8	14.3	6	9.5	14	11.8
Many times	4	7.1	9	14.3	13	10.9
Receive financial help						
Yes	15	26.8	23	36.5	38	31.9
No	41	73.2	40	63.5	81	68.1
Having people helping						
Yes	38	71.7	39	66.1	77	68.8
No	15	28.3	20	33.9	35	31.3
Their relationship with family and friends meets their expectations						
Yes	28	53.9	19	32.2	47	42.3
No	24	46.2	40	67.8	64	57.7

Abbreviation: SD, Standard deviation.

**TABLE 3:** Comparison between control and intervention groups at baseline (based on demand for care, clinical variables and cognitive and functional ability of study subjects)

Variables	Group		Total (*N* = 106), Mean ± SD (range)
	Control (*N* = 51), Mean ± SD	Experimental (*N* = 55), Mean ± SD	
Demand for care			
Visits to the primary care physician	3.27 ± 6.08	3.81 ± 4.18	3.55 ± 3.53 (0–18)
Consultations with the primary care nurse	4.18 ± 6.68	3.56 ± 6.08	3.85 ± 6.39 (0–40)
Domiciliary healthcare	2.66 ± 6.59	1.79 ± 4.90	2.20 ± 5.75 (0–36)
Clinical parameters			
Weight (kg)	71.96 ± 14.84	73.17 ± 12.72	72.61 ± 13.71 (44–106)
Height (cm)	156.53 ± 9.83	157.62 ± 8.59	157.09 ± 9.26 (138–188)
Body Mass Index (BMI)	29.27 ± 4.70	29.19 ± 4.32	29.23 ± 4.48 (18.97–41.23)
Heart Rate (beats/min)	74.06 ± 9.45	73.28 ± 13.23	73.70 ± 11.31 (48–120)
Systolic blood pressure (mm Hg)	132.15 ± 16.97	131.17 ± 14.90	131.70 ± 15.96 (90–178)
Diastolic blood pressure (mm Hg)	72.62 ± 9.37	71.30 ± 8.90	72.01 ± 9.14 (50–95)
Chronic diseases	3.09 ± 2.11	2.98 ± 2.57	2.98 ± 2.35 (0–9)
Functional and cognitive ability			
NOC level of mobility	3.91 ± 5.38	3.66 ± 6.86	3.78 ± 0.84 (1.33–5.00)
NOC depression	3.51 ± 0.70	3.54 ± 0.71	3.53 ± 0.69 (1.82–5.00)
NOC coping	3.30 ± 0.82	3.41 ± 0.72	3.36 ± 0.77 (1.72–5.00)

Abbreviation: SD, Standard deviation.

### 3.2 Effectiveness of the intervention

Regarding the effect of the intervention, no statistically significant difference in the total score of the social isolation variable was found between both groups at the end of follow‐up. However, regarding the specific dimension of confidential support, we found a very close level of statistical significance (*p* = 0.058) according to the intention‐to‐treat analysis (Table 4). These differences are statistically significant in the analysis of this dimension by protocol (*p* = 0.008).

**TABLE 4:** Social isolation. Group comparison between start and end time points

Dimension	Time point 1 (baseline) mean ± SD		Time point 2 (final) mean ± SD		p*	p**	p***
	Control (*N* = 56)	Experimental (*N* = 63)	Control (*N* = 56)	Experimental (*N* = 63)			
Total mean	2.26 ± 0.59	2.26 ± 0.56	2.34 ± 0.70	2.43 ± 0.57	0.565	0.005	0.122
Affective support	1.07 ± 0.26	1.03 ± 0.18	1.12 ± 0.33	1.11 ± 0.32	0.679	0.003	0.096
Confidential support	1.07 ± 0.26	1.02 ± 0.13	1.13 ± 0.33	1.19 ± 0.40	0.058	0.001	0.180
Total support	24.41 ± 6.56	24.90 ± 6.15	25.75 ± 7.78	26.78 ± 7.16	0.565	0.005	0.122

Abbreviation: SD, Standard deviation.

*Global analysis; ANOVA test for repeated measurements; **Analysis between baseline and final measurement in the Experimental group; Wilcoxon test; ***Analysis between baseline and final measurement in Control group; Wilcoxon test.

When analysing the data disaggregated by group, in the experimental one, significant differences were found between the results obtained at baseline (T1) and at the end of the study (T2) in all dimensions. No differences were found in the control group in this regard (Table 4).

However, the total DUFFS (social isolation) scores in the experimental group improved by almost three expected points (scores ranged from 25.00 to 27.94, *p* = 0.005) between the T1 and T2. In addition, the effect of the intervention on this variable was maintained and still improved 2 months after the end of the intervention, that is, at T3 (reaching 28.5, *p* = 0.002 vs. T1).

Concerning the comparison of means associated with the loneliness variable, no statistically significant differences were found between both groups for any of the scores (total, emotional and real) when analysing them at T1 and at T2 (Table 5). However, if we compare paired means in the experimental group, an improvement in emotional loneliness scores between T2 and T3 was found (Wilcoxon test; *p* = 0.012).

**TABLE 5:** Feeling of loneliness. Group comparison between the start and end time points

Dimension	Time point 1 (baseline) mean ± SD		Time point 2 (final) mean ± SD		p*	p**	p***
	Control (*N* = 56)	Experimental (*N* = 63)	Control (*N* = 56)	Experimental (*N* = 63)			
Emotional loneliness	3.95 ± 1.38	2.86 ± 1.73	3.91 ± 1.21	3.02 ± 1.86	0.355	0.331	0.987
Actual loneliness	4.39 ± 1.59	3.63 ± 2.16	4.27 ± 1.70	3.51 ± 2.06	0.992	0.183	0.477
Total loneliness	8.38 ± 2.49	6.51 ± 3.62	8.19 ± 2.32	6.50 ± 3.50	0.588	0.650	0.551

Abbreviation: SD, Standard deviation.

*Global analysis between groups between baseline and final measurements; ANOVA test for repeated measurements; **Analysis between baseline and final measurement in the Experimental group; Wilcoxon test; ***Analysis between baseline and final measurement in Control group; Wilcoxon test.

Regarding HRQOL, the comparison of means of both synthetic indices and health status assessment in both control and experimental groups showed no statistically significant differences.

### 3.3 Associated factors in the improvement of social isolation and loneliness

Table 6 shows the maximum logistic regression model and the most parsimonious final model for the variables associated with the social support of the subjects at the end of the follow‐up period. In this study, the associated variables when adjusted for baseline social support were the number of people helping them (OR = 4.92; 95% CI = 1.15–20.98) and mobility (OR = 3.70; 95% CI = 1.54–8.91), with no significant differences between experimental and control groups (Table 5).

**TABLE 6:** Determinants in the improvement of social isolation

Independent variables	Maximum model				Final model			
	*p*	OR	95% CI of OR		*p*	OR	95% CI of OR	
			Lower limit	Upper limit			Lower limit	Upper limit
Group (experimental vs. control)	0.240	3.33	0.45	240.78	0.911	1.08	0.28	4.13
Age (years)	0.194	1.13	0.938	10.372				
Sex (male vs. female)	0.076	0.05	0.002	10.346				
Marital status: (reference category: widower)								
Married	0.221	30.21	0.12	7,097.37				
Separated	0.816	0.66	0.02	21.15				
Single	0.770	1.59	0.07	36.77				
Education level (reference category: secondary education)								
No studies	0.085	39.96	0.60	2,664.68				
Incomplete primary education	0.212	14.26	0.22	929.19				
Primary school graduate	0.084	69.87	0.57	8,617.74				
Financial difficulties (yes vs. no)	0.095	4.12	0.78	21.77				
Receives financial help (yes vs. no)	0.414	2.32	0.31	17.50				
Medical care (No.)	0.601	0.92	0.69	1.24				
Nurse Care (No.)	0.540	0.94	0.77	1.14				
Domiciliary healthcare (No.)	0.834	1.02	0.83	1.26				
Living alone (yes vs. no)	0.897	0.92	0.28	3.08				
Having people helping (yes vs. no)	0.106	10.64	0.60	187.41	0.031	4.92	1.15	20.98
NOC Mobility	0.006	13.9	2.14	90.35	0.004	3.70	1.54	8.91
NOC depression	0.383	0.31	0.02	4.18				
NOC coping	0.798	1.30	0.17	10.07				
Social support (baseline: yes vs. no)	0.002	222.8	7.56	6,568.41	0.001	35.49	4.60	273.55
Feeling of loneliness (baseline)	0.126	1.40	0.91	2.17				
Health status (Euroqol‐5D)	0.038	0.94	0.89	0.99				
Chronic pathologies (No.)	0.244	0.77	0.50	1.95				

*Note:* Dependent variable: Social support (yes/no). Maximum Model Hosmer‐Lemeshow Test: 0.373. Final Model Hosmer‐Lemeshow Test: 0.856.

Abbreviations: 95% CI, 95% Confidence Interval; OR, Odds Ratio.

Finally, a multivariate analysis was performed to study which independent variables were associated with loneliness; none was statistically significant.

## 4 DISCUSSION

The present study results do not allow us to affirm that the modified CARELINK programme is effective in general terms, although it would be effective in terms of the “confidential support” dimension of social isolation. In addition, it can maintain its beneficial effects 2 months after the intervention.

These results are inconsistent with those obtained by Nicholson and Shellman (2013), who reported a significant improvement in the social isolation of the elderly. The lack of overall effectiveness found in the results of our study may be due to three issues. First, the severity of both social isolation and loneliness of the study population. Regarding social isolation, we found that both groups have low levels of perceived support, with an average value around 24.77; that is also reflected in their affective dimension and in their confidential support dimension separately, with values of 11.27 and 13.30, respectively. In terms of loneliness, the baseline values of both groups are established very close to the mean value of 8.82, which implies an intense level of perceived loneliness. The severe character of both variables could involve a larger difficulty in reaching significant changes. Second, a modified version of CARELINK has been used, which was reduced to make it feasible in professional practice in primary care in our environment. Third, the participating students performed the intervention as volunteers and not as part of their training programme (in contrast with those in the Nicholson & Shellman, 2013). Alongside these reasons, the existence of cultural differences between the two environments could be another reason to be investigated.

Despite this, the results of the study suggest an increase in confidential support, which also continues to improve even after the programme has ended. The reasons for this may be related to the time that each subject needs to acquire the skills developed (dynamizing the communicative and engaging ability of the subject), because resuming the processes of interaction with the social network available around them or creating new links often becomes difficult and requires longer periods (Winningham & Pike, 2007; Chiang et al., 2010).

Regarding the variable loneliness, we found no differences between the experimental and control groups. However, a positive effect on experimental group scores was found (although much less than expected). In addition, this improvement continues over time and even increases after 2 months. This effect appears to be caused by a late improvement in the dimension of emotional loneliness that compensates for worsening the actual loneliness occurring after the intervention ends.

This worsening of actual loneliness can be explained by the effect of the termination of the agent–subject relationship on the perception of the loneliness of that subject. We must bear in mind that this type of intervention starts from the necessary premise of generating an affective link and mutual recognition between the different individuals involved (Nicholson & Shellman, 2013). Thus, the completion of the activity may result in the experience of a duel by the elder people. Regarding the significant improvement in the dimension of emotional loneliness, it may mean that the elder has learned to feel satisfied with the contacts he/she has (remember that the dissatisfaction of expectations with family and friends was a differential baseline characteristic in experimental subjects). This effect was already reported in previous studies (Castro, 2015; Cosco et al., 2014; Nicolaisen & Thorsen, 2017; Savikko et al., 2005).

In any case, the absence of statistically significant differences between the experimental and control groups is within the expected results, as the CARELINK intervention model was developed to intervene in situations of social isolation. However, its effectiveness in loneliness situations had not been demonstrated (Nicholson, 2012). These results show the premise that social isolation and loneliness are two multidimensional phenomena that share elements but at the same time have significant peculiarities (Leigh‐Hunt et al., 2017; Palmer, 2019) that make it necessary to propose objectively differentiated interventions for each of these phenomena (Newall & Menec, 2019) in such a way that, for social isolation, those interventions that make possible social interactions would be more desirable, and in the case of loneliness, psychological reorientation interventions would be more appropriate (Gené‐Badia et al., 2020). Likewise, the literature shows that group interventions are more effective against loneliness than those conducted individually (Findlay, 2003; Cattan et al., 2005; Hagan et al., 2014).

Although the quality‐of‐life outcome data from our study shows no differences between control and experimental groups, several studies reported an improvement that appears to occur as the parameters associated with social isolation and loneliness improve (Kobayashi & Steptoe, 2018). However, although many systematic reviews have identified a significant relationship between social isolation, loneliness and quality of life (Courtin & Knapp, 2017), the direction of this relationship is inconsistent in the literature (Beller & Wagner, 2017).

The results of this study identify the influence of having people that support the elder's and elder's mobility as factors associated with the improvement in social isolation. Both elements are explained because they are necessary conditions, together with communication capacity, to enjoy a dynamic of adequate and satisfactory social interaction (Dykstra, 1995; Rodríguez López & Castro Clemente, 2019; Pinquart & Sorensen, 2001; Savikko et al., 2005).

### 4.1 Limits

First, it is necessary to point out limitations arising from the proposed intervention itself because it aimed to improve social isolation, but it was not specifically designed for situations of unwanted loneliness or quality of life.

Second, it should be noted that the intervention has finally been conducted by nursing students and voluntary staff instead of healthcare professionals who perform their care practice in the primary healthcare system. Therefore, the effect that the initially designed intervention (conducted by healthcare professionals) would have had is unknown.

Third, the sample size achieved was lower than that established initially due to the percentage of subjects who did not complete the study, especially in the experimental arm, which probably caused differential bias and a problem of sufficient statistical power. In relation to the results obtained, we believe that a larger study would provide more conclusive results that would allow us to confirm the effect of the intervention. Therefore, we consider it necessary, similarly to the study performed by Nicholson and Shellman (2013), to continue to study these phenomena with larger sample sizes, and establishing, also, specific and differentiated interventions for each of the phenomena studied, and trying, as far as possible, to place them within the scope of the practice of healthcare professionals. We believe that overcoming these issues would make it possible to discriminate conclusively on the effective nature of the intervention.

Furthermore, it is expected that there would be a “Hawthorne effect” or bias of the observed. This bias is inherent to all experimental studies, resulting even larger when blinding methods cannot be used. However, it was always tried that the participants experienced the intervention as normal and natural (normal conditions), and not as a trial in which we tried to demonstrate its effectiveness (experimental conditions). Moreover, while it is true that, in the early stages of the study, the appearance of this effect is expected, it tends to be neutralized and disappear over time (Gale, 2004). Thus, in a long‐term study, such as the one presented here, the Hawthorne effect would tend to equate control and experimental groups, and to improve the performance of both, but would tend to disappear at the time of measurements (McCarney et al., 2007).

## 5 CONCLUSION

The study results do not show the effectiveness of the modified CARELINK programme, analysed as a whole, on the decrease in social isolation or loneliness or the improvement in HRQL. However, differences in some of the dimensions of these phenomena have been found. The study results suggest an improvement in the “confidential support” of older adults undertaking an intervention through the modified CARELINK programme; and, in the same direction, an improvement in emotional loneliness scores 2 months after the end of the intervention. Regarding the factors associated with the improvement in intervention, the possibility of having people to help them and a larger degree of mobility have been identified as factors favouring the decrease of social isolation.

## AUTHOR CONTRIBUTIONS

Conceptualization: Hernández – Ascanio, José; Perula ‐de Torres, Luis Ángel; RICH – RUIZ, Manuel; Data curation: Hernández – Ascanio, José; (“ASyS study collaborative group”) Formal análisis: Hernández – Ascanio, José; RICH – RUIZ, Manuel; Investigation: Hernández – Ascanio, José; Perula ‐de Torres, Luis Ángel; Methodology: Hernández – Ascanio, José; RICH – RUIZ, Manuel; Project administration: Hernández – Ascanio, José; Perula ‐de Torres, Luis Ángel; RICH – RUIZ, Manuel; Resources: Perula ‐de Torres, Luis Ángel; RICH – RUIZ, Manuel; González‐Santos, Josefa; Mielgo‐Ayuso, Juan; González‐Bernal, Jerónimo; Supervision: Hernández – Ascanio, José; RICH – RUIZ, Manuel; Writing – original draft: Hernández – Ascanio, José; RICH – RUIZ, Manuel; González‐Santos, Josefa; Mielgo‐Ayuso, Juan; González‐Bernal, Jerónimo; Writing – review & editing: Hernández – Ascanio, José; Perula ‐de Torres, Luis Ángel; RICH – RUIZ, Manuel; González‐Santos, Josefa; Mielgo‐Ayuso, Juan; González‐Bernal, Jerónimo.

All authors have agreed on the final version and meet at least one of the following criteria [recommended by the ICMJE (http://www.icmje.org/recommendations/)]:

- substantial contributions to conception and design, acquisition of data or analysis and interpretation of data;
- drafting the article or revising it critically for important intellectual content.

## ETHICAL APPROVAL

The study was performed following the ethical principles of the Helsinki Declaration (Krleza‐Jeric & Lemmens, 2009) and was approved by the Ethics and Clinical Research Committee of the Reina Sofia Hospital in Córdoba (Spain). The participants were informed about the study and signed an informed consent. At all times, the person's anonymity was guaranteed. The personal data obtained have been processed in accordance with Regulation EU/2016/679, of April 27, 2016, General Data Protection, and Organic Law 3/2018, of 5 December, on the Protection of Personal Data and the Guarantee of Digital Rights.

## Data Availability

Data available on request due to privacy/ethical restrictions. The data that support the findings of this study are available on request from the corresponding author. The data are not publicly available due to privacy or ethical restrictions.

## APPENDIX 1

In addition to the authors, the work team that has participated in the research project associated with this article (“ASyS study collaborative group”) is made up of the following people: Ana María Roldán‐Villalobos; Carlos Pérula‐de Torres; Rodrigo Fernández‐Márquez; Caridad Dios‐Guerra; Manuela Urbano‐Priego; Silvia Luna‐Morales; Rafaela Muñoz‐Gómez; Miguel Á. Gómez‐Torres; Ángeles Pastor‐López; Mª José Ibáñez‐Fernández; Mª Dolores Aguilera‐López; Antonio González‐Delgado; Diego Garrido‐Gálvez; Trinidad Romero‐Sánchez; Inmaculada Guzmán‐Castilla; Mª Dolores Maestre‐Serrano; Mari Paz Gutiérrez‐Martín; Eva Sánchez‐Cañete; Yolanda Sánchez‐Palomo; Manuela Rodríguez‐Priego; Ana Mª Pérez‐Trujillo; Santiago Cruz‐Velarde; Sonia Calero‐Sánchez; Rocío Hidalgo‐Navarro; Fco. Javier Ruiz‐Moruno; Mª Reyes Martínez‐Guillén; Antonia Domínguez‐Ramírez; Mª Ángeles Ortega‐Osuna; Paulina Menéndez‐Sagrado; Montserrat Jabalera‐Ramírez; Fernanda Casado‐Salinas; Carmen Mª Mirás‐García; Pilar Conde‐Moya.
